# Cardiac Shock Wave Therapy Alleviates Hypoxia/Reoxygenation-Induced Myocardial Necroptosis by Modulating Autophagy

**DOI:** 10.1155/2021/8880179

**Published:** 2021-01-19

**Authors:** Quan Qiu, Tao Shen, Xiaoxue Yu, Na Jia, Kaiyi Zhu, Que Wang, Bing Liu, Qing He

**Affiliations:** ^1^Department of Cardiology, Beijing Hospital, National Center of Gerontology, Beijing 100730, China; ^2^The Key Laboratory of Geriatrics, Beijing Institute of Geriatrics, Beijing Hospital, National Center of Gerontology, National Health Commission, Institute of Geriatric Medicine, Chinese Academy of Medical Sciences, Beijing 100730, China; ^3^Department of Cardiology, The First Hospital of Shanxi Medical University, Taiyuan, Shanxi 030001, China

## Abstract

Regulated necrosis (necroptosis) is crucially involved in cardiac ischaemia-reperfusion injury (MIRI). The aim of our study is to investigate whether shock wave therapy (SWT) is capable of exerting protective effects by inhibiting necroptosis during myocardial ischaemia-reperfusion (I/R) injury and the possible role of autophagy in this process. We established a hypoxia/reoxygenation (H/R) model *in vitro* using HL-1 cells to simulate MIRI. MTS assays and LDH cytotoxicity assay were performed to measure cell viability and cell damage. Annexin V/PI staining was used to determine apoptosis and necrosis. Western blotting was performed to assess the changes in cell signaling pathways associated with autophagy, necroptosis, and apoptosis. Reactive oxygen species (ROS) production was detected using DHE staining. Autophagosome generation and degradation (autophagic flux) were analysed using GFP and RFP tandemly tagged LC3 (tfLC3). HL-1 cells were then transfected with p62/SQSTM1 siRNA in order to analyse its role in cardioprotection. Our results revealed that SWT increased cell viability in the H/R model and decreased receptor-interacting serine/threonine-protein kinase 1 (RIPK1) and RIPK3 expression. ROS production was also inhibited by SWT. Moreover, SWT decreased Beclin1 expression and the ratio of LC3-II/LC3-I following H/R. Simultaneously, in the tfLC3 assay, the SWT provoked a decrease in the cumulative autophagosome abundance. siRNA-mediated knockdown of p62 attenuated H/R-induced necroptosis, and SWT did not exert additive effects. Taken together, SWT ameliorated H/R injury by inhibiting necroptosis. SWT also relieved the blockade of autophagic flux in response to H/R injury. The restoration of autophagic flux by SWT might contribute to its cardioprotective effect on necroptosis following H/R injury.

## 1. Introduction

Ischaemic heart disease has long been a leading cause of morbidity and mortality worldwide. The current therapeutic options following acute myocardial infarction (AMI), including medical treatment, percutaneous coronary intervention (PCI), and thrombolytic therapy, have focused on the rapid restoration of coronary artery blood flow, which might rescue the viable ischaemic myocardium. However, the rapid reperfusion of the ischaemic myocardium may lead to sustained and even irreversible tissue damage, which ultimately causes myocardial cell death. This phenomenon is referred to as ischaemia/reperfusion (I/R) injury [1]. I/R-induced cell death is now established as an important component of cardiac remodelling, particularly in the transition towards overt heart failure [2]. I/R injury decreases the therapeutic advantage of the aforementioned interventions. Thus, the development of novel therapeutic strategies focusing on limiting reperfusion-induced myocyte death following AMI will increase the salvage of the ischaemic myocardium and is necessary to improve the clinical outcomes in patients with ischaemic heart disease [3]. In this context, cardiac shock wave therapy (CSWT) emerges as a possible candidate.

CSWT is a novel, noninvasive approach that has been shown to ameliorate myocardial ischaemia and improve cardiac function [4, 5]. Previous clinical trials have shown that CSWT improves symptoms, exercise capacity, and myocardial perfusion in patients with severe coronary artery disease [4]. According to the results of animal studies, CSWT ameliorates both myocardial ischaemia and left ventricular remodelling *in vivo* after AMI by upregulating VEGF expression and inducing neovascularization [5]. All these studies describe the beneficial effect of CSWT on ischaemic cardiomyocytes. However, in the current clinical setting, most patients with AMI receive reperfusion therapy. Therefore, an evaluation of the influence of CSWT on myocardial I/R injury would be very meaningful. Importantly, CSWT was shown to ameliorate left ventricular remodelling after myocardial I/R injury in pigs *in vivo* [6]. However, the precise mechanism remains to be elucidated.

The death of cardiomyocytes following I/R injury has been postulated to be mainly caused by apoptosis and necrosis in response to excessive cellular stress [7]. Myocardial apoptosis, which is initiated shortly after ischaemia, is amplified by reperfusion and partially contributes to overall cardiomyocyte death [8]. Blockade of the apoptotic process may minimize cardiac injury induced by I/R, prevent the loss of cardiomyocytes, and slow the occurrence of myocardial stunning and heart failure [9]. As necrosis has long been considered passive and accidental [10], apoptosis was thought to be the only type of cell death in the heart that is possible to manipulate. However, a growing body of evidence has refuted this view and revealed the existence of multiple pathways regulating necrosis [11, 12]. This process is referred to as programmed necrosis or necroptosis and is mediated by RIPK1 and 3 [11, 12]. Necroptosis is prominent in the I/R heart, indicating that necroptosis plays a significant role in the pathological process of myocardial reperfusion injury [13, 14]. Importantly, inhibition of necroptosis was recently shown to alleviate reperfusion injury following AMI in mice, rats, and pigs [15–18]. Therefore, a combination of inhibitors of necroptosis and apoptosis enhances the cardioprotective effect on myocardial I/R injury [16]. The beneficial effects of CSWT on an animal model of I/R injury prompted us to investigate whether it is capable of exerting protective effects on myocardial I/R injury by inhibiting both apoptosis and necroptosis [6].

Autophagy is well known as an intracellular lysosomal self-degradation process that removes and recycles long-lived proteins and damaged organelles to maintain cellular homeostasis. However, excess autophagy is responsible for self-destruction and ultimately causes autophagic cell death, accompanied by the upregulation of LC-3 and Beclin1 [19]. Dysregulated autophagy has recently been suggested to be a potential therapeutic target in the treatment of various cardiac diseases, such as myocardial infarction [20], myocardial I/R injury [21], and cardiac hypertrophy [22]. Based on accumulating evidence, autophagy plays a considerable role in mediating myocardial death following I/R injury [21, 23]. The beneficial effects of inhibition of Beclin1 and the repression of excessive cardiomyocyte autophagy following I/R injury are well established [24]. Accordingly, appropriate interventions that regulate autophagy are anticipated to limit I/R injury and improve cardiac function. However, researchers have not clearly determined whether and how autophagy regulates necroptosis in myocardial cells during reperfusion.

In the present study, we established a hypoxia/reoxygenation (H/R) model *in vitro* using HL-1 cells to determine whether SWT ameliorates myocardial necroptosis and the possible role of autophagy in this process.

## 2. Materials and Methods

### 2.1. Reagents

Dulbecco's modified Eagle's medium, protease inhibitor cocktails (P8340, P5726, and P0044) and the LC3B antibody (Ab) were purchased from Sigma-Aldrich (St. Louis, MO). Trypsin-EDTA, phosphate-buffered saline (PBS), the penicillin/streptomycin antibiotic solution, and FBS were obtained from HyClone (Logan, UT). Lysis buffer, antibodies against AMPK, cleaved caspase-3, Beclin1, GAPDH, p62, p-AMPK, RIPK1, RIPK3, and the HRP-conjugated anti-rabbit IgG secondary antibodies were purchased from Cell Signaling Technology (Beverly, MA). Protein concentrations were determined using a BCA protein assay kit from Pierce (Rockford, AL). The Immobilon Western HRP Substrate was purchased from Millipore (Singapore). The Cell Titer 96® AQueous One Solution Cell Proliferation Assay was obtained from Promega (Madison, WI). The FITC Annexin V Apoptosis Detection Kit (CA1020) and LDH Cytotoxicity Assay Kit (BC0680) were purchased from Solarbio (Beijing, BJ). The MDC kit was purchased from KeyGEN BioTECH (Nanjing, JS). The Lipofectamine RNAiMAX reagent was obtained from Thermo Scientific Invitrogen (Carlsbad, CA). p62/SQSTM1 siRNA (m) was purchased from Santa Cruz Biotechnology (Dallas, TX).

### 2.2. HL-1 Cell Culture

HL-1 cardiomyocytes, a cardiac cell line derived from the AT-1 mouse atrial myocyte tumour lineage, were cultivated in complete DMEM containing 10% (*v*/*v*) foetal bovine serum (FBS), 100 U/mL penicillin, and 100 *μ*g/mL streptomycin. Cells were maintained in a humidified atmosphere with 5% CO_2_ and 95% air (*v*/*v*) at 37°C.

### 2.3. Simulated Hypoxia/Reoxygenation (H/R)

Cells were plated in 60 mm cell culture dishes (Corning, NY). The culture medium was replaced with RPMI-1640 medium lacking FBS when the confluence reached approximately 60-70%. Then, cells were cultured for 24 h to inhibit cell proliferation (synchronization), and hypoxia was induced by a buffer exchange to an ischaemia-mimetic solution (in mM: 125 NaCl, 8 KCl, 1.2 KH_2_PO_4_, 1.25 MgSO_4_, 1.2 CaCl_2_, 6.25 NaHCO_3_, 5 sodium lactate, and 20 HEPES, pH 6.6). Subsequently, the dishes were placed in a hypoxic chamber (HERAcell VIOS 160i, Thermo, USA) equilibrated with 94% N_2_, 5% CO_2_, and 1% O_2_ (*v*/*v*/*v*). After 5 h of hypoxia, reoxygenation was initiated by a buffer exchange to DMEM lacking FBS, and cells were incubated in a chamber containing 95% room air and 5% CO_2_. Controls incubated in normoxic DMEM without FBS were run in parallel for periods that corresponded with the H/R groups.

### 2.4. Shock Wave Treatment

After an incubation under hypoxia conditions for 5 h, cells were subjected to SWT as previously described [25]. After SWT, cells were returned to the incubator with a 95% air and 5% CO_2_ atmosphere and cultured at 37°C for 12 h before harvest.

### 2.5. Cell Viability Assay

Cell viability was estimated using a Cell Titer-AQueous One Solution Cell Proliferation Assay. HL-1 cells were harvested after hypoxia with/without SWT treatment and then seeded in 24-well plates in quadruplicate. HL-1 cells were cultured in normoxic condition for 12 h. Then, 45 microliters of detection reagent was added to each well of the 24-well plates with 225 *μ*L of culture medium. Four hours later, the optical density (OD) was measured at 490 nm.

### 2.6. Cell Cytotoxicity Assay

Lactate dehydrogenase (LDH) is a cellular enzyme that is released upon membrane damage and a recognized marker of cell damage or death. Cell culture supernatants were harvested after H/R, and LDH concentrations were measured using the LDH Cytotoxicity Assay Kit according to the manufacturer's protocol with a routine microtiter plate reader (wavelength: 572 nm).

### 2.7. Monodansylcadaverine (MDC) Staining

MDC staining is a specific method used to detect autophagic vacuoles formed during the process of autophagy. When the adherent HL-1 cells reached the appropriate confluence (50-60%) in 6-well plates, cells were performed according to the manufacturer's protocol. Briefly, 400 *μ*L of MDC working buffer was added into each well, and the cells were incubated in the dark at room temperature for 30 min. HL-1 cells were viewed using a fluorescence microscope at 512 nm and quantified using the ImageJ software (NIH) [26].

### 2.8. GFP and RFP Tandemly Tagged LC3 (tfLC3) Assay

The method used to evaluate tandem fluorescent LC3 puncta following transduction with the mRFP-GFP-LC3 adenovirus was described in a previous study [27]. Briefly, HL-1 cells were transduced with the mRFP-GFP-LC3 adenovirus for 24 h and then treated with H/R or SWT. Then, cells were viewed with an inverted fluorescence microscope.

### 2.9. Western Blotting Analysis

HL-1 cells were harvested as described above, washed twice with ice-cold PBS, and lysed in ice-cold lysis buffer supplemented with phosphatase and protease inhibitors. Afterwards, total cell lysates were incubated on ice for 5 min and centrifuged at 13000 g for 15 min (4°C), and protein concentrations of the supernatants were determined using a Pierce BCA Protein Assay Kit. Twenty micrograms of soluble proteins was electrophoresed on 12% SDS polyacrylamide gels and transferred to polyvinylidene fluoride (PVDF) membranes that were then blocked with TBS-T buffer (0.15 M NaCl, 0.05 M Tris hydroxymethyl methylamine, and 0.1% Tween-20, pH 7.4-7.6) containing 5% nonfat milk for 2 h at room temperature. Membranes were washed three times with TBS-T buffer and incubated overnight at 4°C with specific primary Abs (1 : 1000). Membranes were then washed three times with TBS-T buffer and incubated with secondary Ab (1 : 5000) for 2 h at room temperature, followed by washes as described above. Then, the immunoreaction was visualized with an ECL detection reagent and quantified using the ImageJ software (NIH).

### 2.10. Flow Cytometry

The percentages of apoptotic cells were analysed using flow cytometry after fluorescein isothiocyanate-conjugated Annexin V (FITC-Annexin V) and propidium iodide (PI) staining. The staining procedure was performed according to the instructions provided with the Annexin V-FITC Apoptosis Detection Kit. HL-1 cells in the early logarithmic growth phase were seeded in 6-well plates, harvested by centrifugation at 800 g for 5 min, and then washed twice with PBS. Cells were resuspended in 100 *μ*L of 1x binding buffer to a density of 1∗10^6^ cells/mL, 5 *μ*L of Annexin V-FITC was added, and the cells were incubated in the dark at room temperature for 10 min. Then, 5 *μ*L of PI was added, and the cells were incubated in the dark at room temperature for another 5 min. Five hundred microliters of 1x binding buffer was added to the mixture, which was then loaded onto a flow cytometer (FACSCalibur, BD, USA) for the apoptosis analysis.

### 2.11. Cell Transfection

HL-1 cells were transfected with SQSTM1 siRNA (10 *μ*M, similarly hereinafter) or negative controls using the Lipofectamine RNAiMAX reagent according to the manufacturer's instructions. First, SQSTM1 siRNA was diluted with a siRNA transfection medium (50 nM) [28]. Then, HL-1 cells were exposed to siRNA mixture for 6 hours, following which transfection medium was replaced with fresh complete media. After 24 h from transfection, cells were treated with hypoxia and SWT.

### 2.12. DHE Staining

HL-1 cells were stained with 10 *μ*mol/L dihydroethidium (DHE, Sigma) in a dark, humidified chamber at 37°C for 30 min to evaluate the ROS level. ROS generation was indicated by red fluorescence detected with a fluorescence microscope and quantified using the ImageJ software.

### 2.13. Statistical Analysis

Results are presented as means ± SEM. Statistically significant differences were assessed with unpaired 2-tailed Student's *t*-test for two experimental groups and one-way ANOVA for multiple groups using SPSS software. Bonferroni's post hoc test was employed after ANOVA to determine significant differences between groups. A probability value of less than 0.05 was considered statistically significant.

## 3. Results

### 3.1. SWT Increased Cell Viability and Attenuated the Cytotoxicity of HL-1 Cardiomyocytes Exposed to H/R

**Figure 1:**
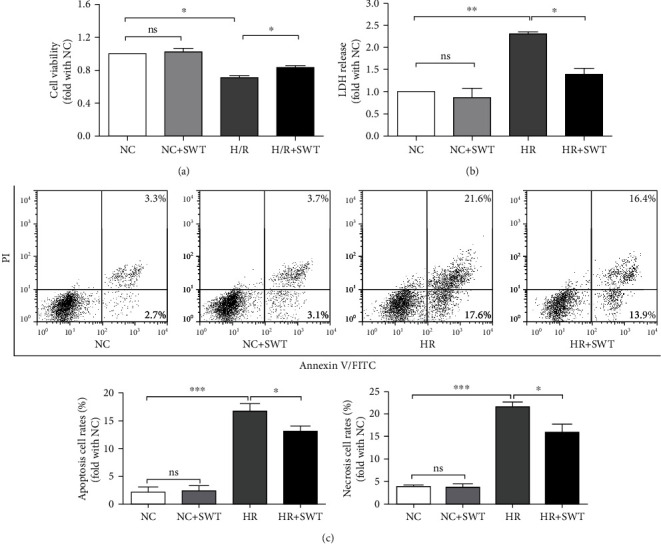
SWT increased cell viability and attenuated the cytotoxicity of HL-1 cardiomyocytes exposed to H/R. (a) The cell viability was measured using the MTS assay in HL-1 cells treated with/without H/R and SWT (*n* = 5). (b) LDH concentration in the medium after exposure to H/R and/or SWT (*n* = 3). (c) Annexin V/PI double staining was used to assess the apoptosis and necrosis of HL-1 cells treated with/without H/R and SWT (*n* = 4). ^∗^*P* < 0.05, ^∗∗^*P* < 0.01, and ^∗∗∗^*P* < 0.001. ns: not significant.

The HL-1 cell line is acknowledged as an excellent model for studying the physiological features of cardiac cells [29]. The *in vitro* HL-1 cell H/R model better resembles cardiac I/R injury *in vivo* [30, 31]. Using the MTS method to monitor the viability of HL-1 cells, HL-1 cell viability was significantly decreased after a 5 h incubation under hypoxic conditions followed by 12 h of reoxygenation, which induces a moderate degree of cell injury. We subjected the HL-1 cells to the H/R treatment and applied SWT to cells during the first 10 min of reoxygenation to determine whether SWT exerted a cardioprotective effect through a direct action on cardiomyocytes. We assessed cell viability with the MTS assay and found that SWT substantially increased cell viability after the H/R treatment (Figure 1(a)). H/R induced a significant increase in LDH leakage, another index of cellular damage, into culture supernatants, which was decreased by the SWT treatment (Figure 1(b)). Based on our findings, SWT increased cell viability and attenuated the cytotoxicity of HL-1 cardiomyocytes exposed to H/R.

### 3.2. SWT Inhibited the Apoptosis and Necrosis of HL-1 Cells Induced by H/R Injury

**Figure 2:**
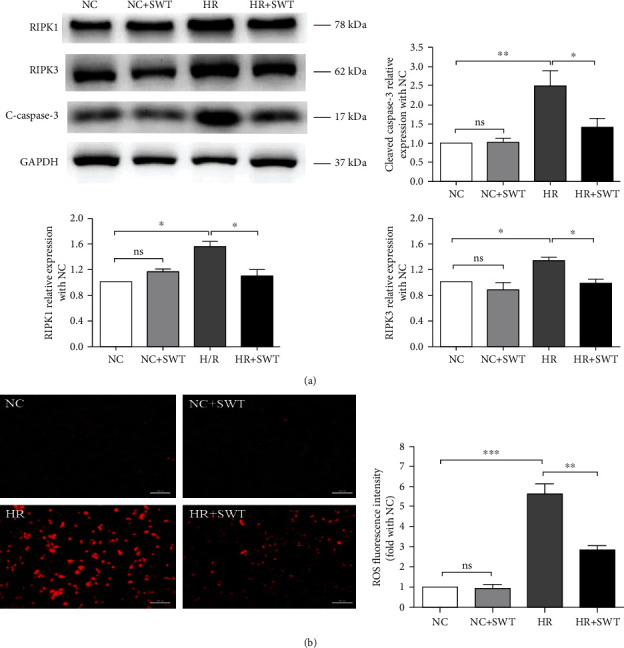
SWT inhibited the apoptosis and necroptosis of HL-1 cells and decreased ROS production induced by H/R injury. (a) Western blotting analysis showing cleaved caspase-3 (c-caspase-3), RIPK1, and RIPK3 levels in HL-1 cells treated with/without H/R and SWT (*n* = 3). (b) DHE staining for HL-1 cells treated with/without H/R and SWT (*n* = 4). ^∗^*P* < 0.05, ^∗∗^*P* < 0.01, and ^∗∗∗^*P* < 0.001. ns: not significant.

Annexin V/PI double staining was used to assess the percentages of apoptotic and necrotic HL-1 cells. The percentage of apoptotic cells significantly increased from 2.16 ± 0.61% in the NC group to 16.73 ± 0.77% in the H/R group, and the percentage of necrotic cells increased from 3.55 ± 1.12% to 21.63 ± 0.61%, suggesting that both apoptosis and necrosis are involved in cardiomyocyte death induced by I/R injury. Then, we quantified the percentages of apoptotic and necrotic cells in the SWT-treated H/R group and found that SWT decreased the percentage of apoptotic cells to 13.12 ± 0.63% and the percentage of necrotic cells to 15.83 ± 1.16% in the H/R group (Figure 1(c)). The levels of the apoptosis-related protein cleaved caspase-3 were then detected using Western blotting. H/R exposure induced apoptosis by increasing the levels of cleaved caspase-3. Consistent with our flow cytometry results for the apoptosis level, SWT markedly reversed the effect on the level of cleaved caspase-3 in the cells exposed to H/R. The H/R+SWT group exhibited lower levels of cleaved caspase-3 than the H/R group (Figure 2). Therefore, both apoptosis and necrosis induced by H/R injury were attenuated by SWT.

### 3.3. SWT Inhibited Necroptosis and ROS Production in HL-1 Cells Induced by H/R Injury

Necroptosis is now considered a subtype of necrosis and recognized as an important contributor to necrotic damage in cardiac I/R injury [15–18]. Necroptosis is reported to be regulated by both RIPK1 and RIPK3 [32]. Then, the levels of RIPK1 and RIPK3 were assessed using Western blotting to observe the effects of H/R on necroptosis. The levels of both RIPK1 and RIPK3 were significantly increased in the H/R group, indicating that both proteins were activated by H/R injury and contributed to cardiac necroptosis. We next evaluated the impact of SWT on necroptosis in response to H/R injury by determining the levels of both proteins. The administration of SWT to the H/R group decreased RIPK1 and RIPK3 levels compared to the H/R group (Figure 2). As reported previously, ROS are implicated in the process of RIPK3-induced necroptosis in cardiac I/R injury [33]. Therefore, we determined whether SWT inhibited ROS production in the H/R model. Using DHE staining, we revealed that H/R induced ROS production and SWT reversed it (Figure 2(b)). Based on our data, H/R-mediated necroptosis and ROS production were inhibited by the SWT.

### 3.4. SWT Restored Autophagy in H/R-Treated Cardiomyocytes

**Figure 3:**
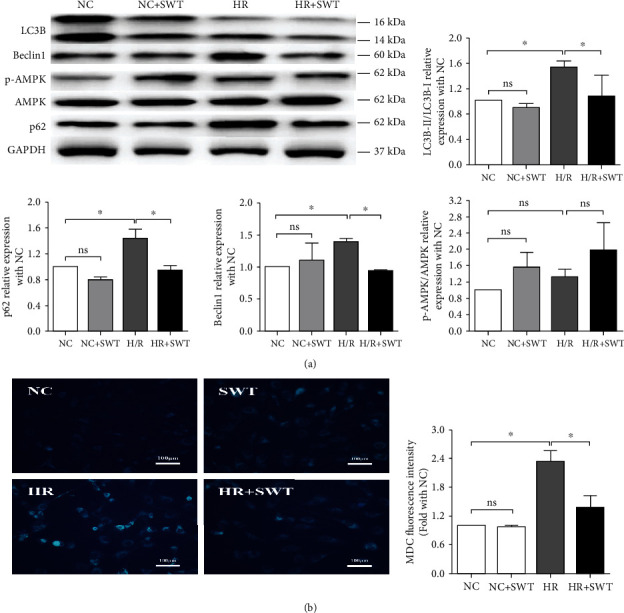
H/R enhanced autophagy in HL-1 cardiomyocytes, and SWT inhibited the autophagy of HL-1 cells induced by H/R injury. (a) Western blotting and average levels of LC3B II/I, p62, Beclin1, p-AMPK, and AMPK in the NC, NC+SWT, H/R, and H/R+SWT groups (*n* = 3). (b) Images of MDC staining and quantitative analysis of the average fluorescence intensity of the autophagic vacuoles in the NC, NC+SWT, H/R, and H/R+SWT groups (*n* = 3). ^∗^*P* < 0.05, ^∗∗^*P* < 0.01, and ^∗∗∗^*P* < 0.001. ns: not significant.

The cytosolic form of LC3 (LC3-I) undergoes lipidation to form LC3-II after the induction of autophagy, and LC3-II is subsequently recruited to the autophagosomal membrane [34]. Therefore, an increase in the LC3-II/LC3-I ratio is a hallmark of autophagy, which represents an increased number of autophagosomes. H/R led to a significant increase in the LC3-II/LC3-I ratio from its basal level in the NC group. As both phospho-AMPK (p-AMPK) and Beclin1 have been reported to play vital roles in autophagy, we next determined changes in their levels. Beclin1 levels were significantly increased in the H/R group compared with the NC group, while no significant change in the level of p-AMPK was observed. Thus, autophagosome formation in the H/R model was accompanied by the activation of Beclin1, but not p-AMPK. p62/SQSTM1 is another important protein substrate of autophagy and the hallmark of autophagic flux, which links ubiquitinated aggregates for destruction within autophagosomes and is degraded upon autophagosome processing. Intact autophagic flux is always accompanied by a decrease in p62/SQSTM1 levels. However, the expression of p62/SQSTM1 was markedly increased in the H/R group. The accumulation of p62/SQSTM1 in cardiomyocytes subjected to H/R implicated impaired autophagic flux during H/R injury with decreased autophagosome degradation. Meanwhile, the increased expression of p62 in the H/R group was attenuated by the SWT treatment, indicating that SWT reversed the H/R-induced blockade of autophagic flux (Figure 3(a)). Furthermore, MDC was used to label autophagosomes. After exposure to H/R, the number of MDC-positive vacuoles was significantly increased in cardiomyocytes. Interestingly, the SWT significantly decreased the number of MDC-positive vacuoles in cardiomyocytes subjected to H/R injury (Figure 3(b)).

**Figure 4:**
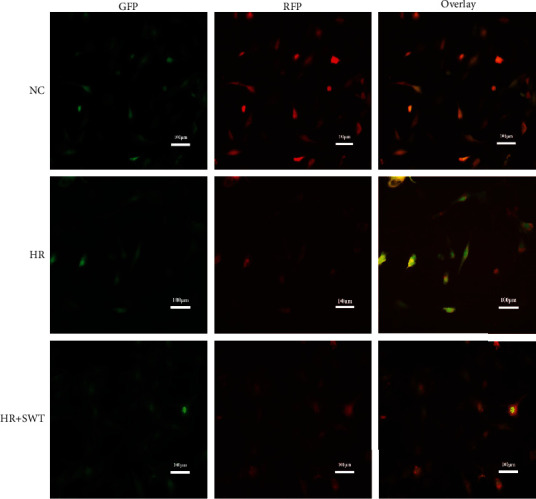
SWT relieved the blockade of autophagic flux induced by H/R injury. HL-1 cells were transfected tandem fluorescent LC3 puncta using the mRFP-GFP-LC3 adenovirus for 24 h prior to exposure to H/R and SWT. Cells were viewed and imaged with an inverted fluorescence microscope (scale bar: 100 *μ*M). The transfection efficiency of tfLC3 was approximately 80-90% after 24 h.

We adenovirally transduced mRFP-GFP tandem-tagged LC3 (tfLC3) to assess the relative abundance of autophagosomes and autolysosomes as a measure of autophagic flux, as described in a previous study [35]. HL-1 cells cultured under normoxic conditions exhibited a preponderance of autolysosomes and a few autophagosomes. H/R exposure resulted in the accumulation of autophagosomes and a decrease in autolysosomes, indicating impaired autophagosome clearance (Figure 4). This result was consistent with the accumulation of the autophagosome-bound proteins LC3-II and p62. According to the results of the tfLC3 assay, the SWT treatment decreased the cumulative autophagosome abundance, suggesting that these vesicles were consumed during autophagy, and markedly increased the abundance of autolysosomes compared with the H/R group, indicating that SWT improved autophagic flux (Figure 4). Thus, the blockade of autophagic flux induced by H/R injury was reversed by SWT.

### 3.5. siRNA-Mediated Knockdown of p62/SQSTM1 Attenuated Necroptosis during H/R Injury in HL-1 Cells, and SWT Did Not Exert Additive Effects

**Figure 5:**
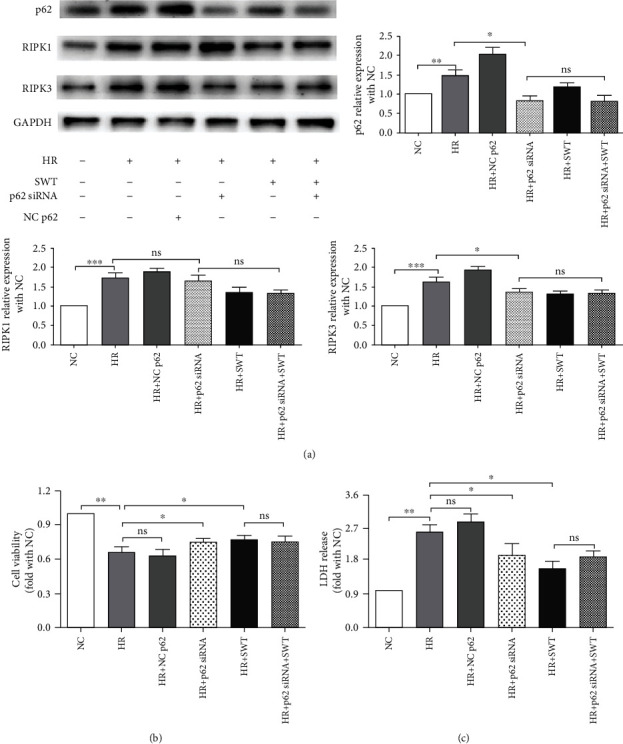
siRNA-mediated knockdown of p62/SQSTM1 attenuated necroptosis during H/R injury in HL-1 cells. (a) Western blotting showing the levels of p62 and RIPK3 in H/R-treated and H/R+SWT-treated HL-1 cells that were pretreated with p62/SQSTM1 siRNA (*n* = 4). (b, c) Effect of p62/SQSTM1 siRNA on cell viability of HL-1 cells exposed to H/R and SWT, as assessed using the MTS assay and LDH cytotoxicity assay (*n* = 4). ^∗^*P* < 0.05, ^∗∗^*P* < 0.01, ^∗∗∗^*P* < 0.001. ns: not significant.

To determine whether there is a relationship between H/R-induced necroptosis and autophagy in cardiomyocytes, p62/SQSTM1 siRNA was transfected to decrease the expression of p62/SQSTM1 in HL-1 cells before H/R treatment.  Figure 5(a) showed that H/R-induced increase in RIPK3 expression was inhibited by siRNA-mediated knockdown of p62/SQSTM1 and the SWT treatment did not further decrease it. Moreover, cell viability was slightly increased, and LDH leakage was attenuated in the H/R+p62/SQSTM1 knockdown group compared with the H/R group, respectively (Figures 5(b) and 5(c)). Taken together, H/R-induced p62/SQSTM1 accumulation was responsible for the necroptosis-mediated death of HL-1 cells following H/R injury, and the protective effects of SWT on H/R-induced necroptosis are mediated by the restoration of autophagic flux.

## 4. Discussion

In the present study, SWT protected against H/R injury in HL-1 cardiomyocytes by inhibiting necroptosis. SWT reversed H/R-induced autophagic flux impairments in autophagosome processing. The restoration of autophagy by SWT contributes to its cardioprotective effect on necroptosis following H/R injury.

Necrotic cell death is an important hallmark of I/R injury [36]. A form of regulated and programmed necrosis called necroptosis was recently identified [37]. Importantly, *in vivo* studies have confirmed that necroptosis contributes to the pathological process of myocardial reperfusion injury, and the inhibition of necroptosis has been shown to alleviate cardiac reperfusion injury [13–18, 33]. The best described signalling cascade that induces necroptotic death in various types of cells requires the RIPK1-RIPK3 complex [38]. Cardiac I/R injury has recently been shown to induce ROS production, resulting in cardiomyocyte necroptosis [33]. Currently, the expression levels of RIPK1 and RIPK3 are utilized as the criteria to evaluate the degree of necroptosis. In the present study, H/R increased RIPK1 and RIPK3 levels in HL-1 cells. Notably, the levels of these molecules were significantly decreased in the H/R+SWT group, indicating that H/R-induced necroptosis was attenuated by the SWT treatment. Thus, SWT inhibited H/R-triggered cardiac necroptosis.

Autophagy is an important intracellular degradation process characterized by the lysosome-dependent turnover of damaged proteins and organelles that plays an important role in maintaining normal cell phenotype and function [39]. However, autophagy is a double-edged sword that may exert both protective and harmful effects. Autophagy promotes cell survival during cardiac ischaemia/hypoxia [40]. On the other hand, autophagy induces cell death via the excessive degradation of essential cellular components when pathological stress induces autophagy dysfunction [40, 41]. Autophagy has recently been reported as a novel regulatory target to limit H/R injury of cardiomyocytes [42, 43]. In the present study, H/R markedly increased autophagosome formation by upregulating the expression of Beclin1 and impairing autophagosome clearance, as evidenced by the accumulation of p62 and the decrease in the number of autolysosomes. Based on this finding, H/R induced autophagy dysfunction and autophagosome accumulation, consistent with the results from the recent study by Ma et al. reporting that H/R impaired autophagic flux and reduced the clearance of autophagosomes [41]. Furthermore, in the present study, SWT restored the autophagic flux in cardiomyocytes during H/R injury and exerted cardioprotective effects.

A previous study has described the interplay between autophagy, apoptosis, and necroptosis during H/R injury [44]. Defective autophagic flux might promote necroptosis through p62-mediated RIPK1 activation [45]. Based on the aforementioned studies, a tempting speculation is that the interaction between p62 and RIPK1 might play an essential role in the induction of necroptosis by blockading autophagy following H/R injury. In the current study, H/R-induced p62/SQSTM1 accumulation was a result of the blockade of autophagic flux in HL-1 cells, which could be markedly ameliorated by SWT. siRNA-mediated knockdown of p62/SQSTM1 attenuated necroptosis during H/R injury in HL-1 cells. Then, we measured the necroptosis level of HL-1 cardiomyocytes cultured under H/R conditions with or without SWT treatment after knocking down p62/SQSTM1 to further clarify the role of autophagy in the cardioprotective effects of SWT. The addition of the SWT treatment did not have additive effects on suppressing necroptosis after p62/SQSTM1 inhibition, suggesting that SWT protected against necroptosis during H/R injury by modulating autophagy. Collectively, the restoration of autophagy by SWT contributes to its cardioprotective effect on necroptosis following H/R injury. Interestingly, cell viability was lower in the HR+p62/SQSTM1 group compared with the HR+SWT group. Therefore, the additional cytoprotective effect by SWT on viability might be mediated by other mechanisms, which required further investigation.

## 5. Conclusion

In conclusion, SWT exerts a protective effect on H/R injury by ameliorating necroptosis. SWT also relieved the blockade of autophagic flux in response to H/R injury. The cardioprotective effects of SWT on necroptosis may be mediated by the restoration of autophagy. SWT may serve as an effective therapeutic option for the prevention of I/R injury in patients with AMI.

## Data Availability

The datasets used and/or analysed during the current study are available from the corresponding author on reasonable request.
